# Course trajectories of anxiety disorders: Results from a 6-year follow-up in a general population study

**DOI:** 10.1177/00048674211009625

**Published:** 2021-04-22

**Authors:** Simone ME Schopman, Margreet ten Have, Anton J van Balkom, Ron de Graaf, Neeltje M Batelaan

**Affiliations:** 1 Department of Psychiatry, Amsterdam Public Health Institute, Vrije Universiteit Amsterdam and Amsterdam UMC, Amsterdam, The Netherlands; 2 GGZ inGeest Specialized Mental Health Care, Amsterdam, The Netherlands; 3 Netherlands Institute of Mental Health and Addiction, Utrecht, The Netherlands

**Keywords:** Anxiety disorders, course, transition, trajectory

## Abstract

**Objective:**

Little is known about the course of anxiety disorders in the general population. This study provides insights into the course of anxiety disorders in the general population taking into account transition to residual symptoms and to other diagnostic categories.

**Methods:**

Using data from three waves of the Netherlands Mental Health Survey and Incidence Study-2 (NEMESIS-2; *n* = 6646), subjects with anxiety disorders (T_0;_ *n* = 243) were divided into three mutually exclusive course trajectories according to their diagnostic status at 3-year (T_1_) and 6-year (T_2_) follow-up: remission group (no disorder at T_2_), intermittent course group (no disorder at T_1_ and disorder at T_2_) and chronic course group (disorder at all measurements). Transition to residual symptoms or other psychopathology were studied. In addition, predictors of course trajectories were assessed.

**Results:**

During 6-year follow-up, 77.8% of subjects achieved remission, 14.0% followed an intermittent course and 8.2% a chronic course. Of those in remission, residual anxiety symptoms remained in 46.6%, while 7.9% developed another disorder between T_0_ and T_2_. Compared with the remitting group, a chronic course was predicted by not living with a partner, multiple negative life events, neuroticism, lower mental functioning, severity of anxiety symptoms, use of mental health care and medication use.

**Limitations:**

The intermittent and chronic course groups were small, limiting statistical power. As a result, certain predictors may not have reached significance.

**Conclusions:**

In the general population at 6-year follow-up, 77.8% of subjects with anxiety disorders achieved remission. Because of transition to residual symptoms or another diagnostic category, only 52.4% of those subjects had a true favourable outcome.

## Introduction

Anxiety disorders have major implications on individual lives and society, accompanied by significant personal and societal costs (Remes et al., 2016; Saarni et al., 2007). In patients seeking treatment, the course of anxiety disorders is characterized by fluctuating symptom levels (Batelaan et al., 2014), high relapse rate following remission (Ansell et al., 2011) and chronicity (Spinhoven et al., 2016). However, for various reasons this picture may be too optimistic. First, remission is usually defined as ‘absence of a *DSM*-anxiety disorder’, irrespective of the presence of residual symptoms. Residual symptoms, however, may reduce quality of life (Haller et al., 2014), are associated with a decreased level of functioning (Batelaan et al., 2007a; Bosman et al., 2019; Fehm et al., 2008) and are accompanied by high costs due to medical costs and absenteeism (Batelaan et al., 2007b). Consequently, residual symptoms contribute to the burden for individuals and society. Second, the occurrence of a disorder from another diagnostic group during the course of an anxiety disorder is hardly ever taken into account in previous research (Hasin and Grant, 2015; Möller et al., 2016; Walsh et al., 2017). In research in which such transitions are studied, the outcome of the index disorder is far less positive, due to the development of other psychopathology. For example, Scholten et al. found that 23.8% of remitted anxiety disorder patients had an anxiety disorder after 4 years. However, more than half of the patients (54.8%) fulfilled criteria of an anxiety or depressive disorder (Scholten et al., 2016).

To date, most studies examining course trajectories have been conducted in clinical populations while few studies have been conducted in the general population. However, results of clinical studies cannot be straightforwardly applied to the general population, since the clinical population can be seen as a subgroup of the general population and population characteristics influence the course critically. For example, it is likely that patients in a clinical setting suffer from more severe and more chronic anxiety disorders in comparison to persons with anxiety disorders in the general population. The few studies that have been conducted in the general population indeed report a more favourable course of anxiety disorders than clinical studies. The Lundy study (Grasbeck et al., 1998) showed that 64.5% of subjects with an anxiety disorder at baseline remitted during a disease course of 25 years. The National Epidemiologic Survey on Alcohol and Related Conditions (NESARC) study showed 66.9% spontaneous remission of anxiety disorder after 3 years (Henriksen et al., 2015). The Longitudinal Aging Study Amsterdam (LASA) study (Schuurmans et al., 2005) in an elderly population found 77% of subjects without an anxiety disorder at 6-year follow-up. And in the Netherlands Mental Health Survey and Incidence Study-1 (NEMESIS-1) with naturalistic follow-up within the Netherlands Study of Depression and Anxiety (NESDA) study (Rhebergen et al., 2011), 46.5% of the subjects with an anxiety disorder at baseline were free of symptoms during 7-year follow-up. However, a comprehensive picture of the course of anxiety disorders in the general population is lacking, because residual symptoms and the development of psychopathology other than the index disorder are typically not studied.

The Netherlands Mental Health Survey and Incidence Study-2 (NEMESIS-2) is a prospective psychiatric epidemiological study among the general population. Using data of three waves of this study, we examined (1) the course of anxiety disorders, including social anxiety disorder (SAD), panic disorder (PD) with or without agoraphobia, agoraphobia (AG) and generalized anxiety disorder (GAD) in the general population over a 6-year follow-up period, divided into remitting, intermittent and chronic course; (2) in the remitting group, the presence of a disorder from a diagnostic group other than those of the anxiety disorders; (3) presence of subthreshold symptoms in the remitting group; and (4) factors that predict an unfavourable course in the general population, including intermittent or chronic course.

## Methods

### NEMESIS-2

The Netherlands Mental Health Survey and Incidence Study-2 (NEMESIS-2) is a psychiatric epidemiological cohort study of the Dutch general population aged 18–64 years at baseline. It is based on a multistage, stratified random sampling of households, with one respondent randomly selected in each household. The face-to-face interviews were computer-assisted. In the first wave (T_0_), performed from November 2007 to July 2009, 6646 persons were interviewed (response rate 65.1%). This sample was nationally representative, although younger subjects were somewhat underrepresented (de Graaf et al., 2010). Three years after T_0_, 5303 persons were able to be re-interviewed (response rate 80.4%) (de Graaf et al., 2013a). Three years after T_1_, 4618 persons were interviewed again (response rate 87.8%) (de Graaf et al., 2013b). Attrition between T_0_ and T_2_ was not significantly associated with all 12-month mental disorders at T_0_ after controlling for sociodemographics, except for bipolar disorder (de Graaf et al., 2013a). The study was approved by a medical ethics committee. After having been informed about the study aims, respondents provided written informed consent at each wave. For a more comprehensive description of the design, see de Graaf et al. (2010).

### Sample

In the current study, subjects with a 12-month anxiety disorder according to the Composite International Diagnostic Interview (CIDI) at baseline (T_0_) who participated in all follow-up assessments were included. Anxiety disorders included panic disorder with or without agoraphobia, agoraphobia without panic disorder, social phobia and generalized anxiety disorder. A comorbid mood disorder or substance use disorder at baseline was allowed, but those with lifetime bipolar disorder or schizophrenia at baseline were excluded (*n* = 31). The final sample consisted of 243 subjects.

### Diagnostic instrument

Psychiatric disorders according to *Diagnostic and Statistical Manual of Mental Disorders*, fourth edition (*DSM*-IV) were diagnosed using the CIDI version 3.0, a fully structured lay-administered diagnostic interview. The CIDI 3.0 version used in NEMESIS-2 was an improvement on the Dutch version used in the World Mental Health Survey Initiative (Kessler and Üstün, 2004). Clinical calibration studies in various countries (Haro et al., 2006) found that the CIDI 3.0 assesses mood, anxiety and substance use disorders with generally good validity and reliability in comparison to blinded clinical reappraisal interviews. The CIDI assesses a lifetime diagnosis and a 12-month diagnosis. In this study, we included subjects with a 12-month anxiety disorder diagnosis at T_0_. The lifetime framework of the CIDI at T_1_ and T_2_ was adapted to the 3-year T_0_–T_1_ and T_1_–T_2_ intervals.

### Course

Three course types were distinguished: remitting, intermittent course and chronic course (see Table 1). All subjects had a 12-month anxiety disorder at baseline. The remitting group consisted of subjects without a 3-year diagnostic status of an anxiety diagnosis at T_2_. The intermittent course group consisted of subjects with a 3-year diagnostic status of anxiety disorder at T_2_, but without a 3-year diagnostic status at T_1_. The chronic course group consisted of subjects with a 3-year diagnostic status of an anxiety disorder at T_1_ and T_2_. We did not differentiate between the separate anxiety disorders, since diagnostic instability between the different separate anxiety disorders is rather high (Hovenkamp-Hermelink et al., 2016; Scholten et al., 2016; Wittchen et al., 2000), comorbidity between anxiety disorders frequently occurs and to warrant sufficient statistical power. Thus, a subject with, for example, panic disorder at T_0_ and GAD at T_1_ and T_2_ was assigned to the ‘chronic course’ group.

**Table 1.** Definitions of remitting, intermittent and chronic course.

	T_0_	T_1_	T_2_
Remitting course	Anxiety diagnosis	Anxiety diagnosis or no diagnosis	No diagnosis
Intermittent course	Anxiety diagnosis	No diagnosis	Anxiety diagnosis
Chronic course	Anxiety diagnosis	Anxiety diagnosis	Anxiety diagnosis

### Subthreshold symptoms

Subjects who remit from an anxiety disorder may experience residual anxiety symptoms. As there is currently no consensus on the definition of subthreshold symptoms in anxiety disorders, we used the definitions described below for the specific subthreshold disorders, following Bosman et al. (2019). For subthreshold panic disorder, respondents experienced ⩾1 unexpected panic attacks of at least mild severity with or without interference. For subthreshold social phobia and subthreshold agoraphobia, respondents experienced social phobia or agoraphobia symptoms of at least mild severity in ⩾1 situations with or without panic symptoms and with or without interference. For subthreshold GAD, respondents experienced >1 worries for at least 1 month with at least mild severity with or without interference.

### Transition to other diagnostic categories

In the remitting group (no anxiety disorder at T_2_), transition to other diagnostic categories was defined as presence of a 3-year diagnosis of a mood or substance use disorder at T_2_ which was not present at T_0_, while the anxiety disorder was in remission at T_2_. For example, when a subject had panic disorder and no other mental disorders at T_0_ and did not meet criteria for any anxiety disorder at T_2_, but met criteria for a 3-year mood disorder at T_2_, this subject made a transition to ‘another diagnostic category’.

### Putative predictors

We assessed sociodemographic, vulnerability and clinical factors as putative predictors for a poor course trajectory, including intermittent and chronic course trajectory, as compared with the remitting trajectory. All predictors were assessed at T_0_, except for parental psychopathology and conscientiousness which were assessed at T_1_.

Sociodemographic factors included gender, age (18–24/25–34/35–44/45–54/55+ years old), education (no education or primary education/pre-vocational secondary education/secondary vocational or pre-university education/higher professional education or university education), living with a partner (yes/no) and having a job (yes/no).

Vulnerability factors included childhood abuse, number of negative life events, parental psychopathology, number of comorbid somatic disorders, neuroticism, extroversion and conscientiousness. Childhood abuse was defined as having experienced emotional neglect, psychological abuse or physical abuse on ⩾2 occasions, or sexual abuse on ⩾1 occasion before age 16. Negative life events were measured according to presence of ⩾1 of 10 negative life events in the previous 12 months, such as death of a relative or friend, divorce and financial difficulties, based on Brugha et al. (1985); as well as the number of these events. Parental psychopathology was defined as being present if a biological parent had ever been treated by a psychiatrist, or hospitalized in a mental institution, or exhibited one or more of the following problems: severe depression, delusions or hallucinations, severe anxiety or phobias, alcohol abuse, drug abuse, regular problems with the police and suicidal behaviour. Somatic disorders were measured by the presence of ⩾1 of 17 chronic physical disorders treated or monitored by a medical doctor in the previous 12 months, assessed using a standard checklist as well as the number of these disorders. Functioning was based on the Medical Outcomes Study Short-Form Health Survey (SF-36) (Ware and Sherbourne, 1992). The eight subscales were combined into two scales: physical functioning (general health, physical health, physical functioning and bodily pain; α = 0.82) and mental functioning (psychological health, psychological functioning, social functioning and vitality; α = 0.80), ranging from 0 (low) to 100 (high functioning). Neuroticism and extroversion were measured using 12 items (0 =low neuroticism/extraversion; 12 = high neuroticism/extraversion) from the Eysenck Personality Questionnaire–Revised Short Scale (Eysenck et al., 1976, 1985; Sanderman et al., 2012). Conscientiousness was measured using a scale of the NEO Five Factor Inventory (NEO-FFI), with 12 items, each with five response categories (1 =totally disagree; 2 = disagree; 3 = neutral; 4 = agree; 5 = totally agree) (Costa and McCrae, 1992).

Various clinical factors were assessed. Age of onset of the earliest anxiety disorder was assessed at baseline. The diagnosis of the anxiety disorder and the comorbid disorders (major depressive disorder [MDD], dysthymia, alcohol use disorder) were assessed using CIDI data at all waves. Severity of the anxiety disorder was defined as severe self-reported impairment in at least two of four areas of role functioning, as assessed using the Sheehan Disability Scale (SDS) (Leon et al., 1997). Use of psychotropic medication was assessed in the 12 months before baseline and included antidepressants or benzodiazepines, prescribed by a health care professional. Service use refers to at least one contact made in general medical or mental health care for emotional or substance use problems in the 12 months before baseline.

### Statistical analysis

First, those with a 12-month anxiety disorder at baseline were described using descriptive analyses (Table 2). Second, based on the presence or absence of anxiety disorder at T_1_ and T_2_ and according to the definitions mentioned above, subjects were divided into three course trajectories: remitting, intermittent and chronic. Third, transition to a disorder of another diagnostic group and presence of subthreshold symptoms were assessed in the remitting group at T_2_. Fourth, multinomial logistic regression analyses adjusted for gender and age were performed to examine risk indicators for an intermittent and chronic course compared with a remitting course (Table 3). To examine whether risk indicators differed between an intermittent and chronic course, intermittent course was also used as reference group. Analyses were performed using STATA 12.1. Two-tailed testing procedures were used with 0.05 alpha levels in all analyses.

**Table 2.** Baseline description of the study group consisting of subjects with any anxiety disorder at baseline (*n* = 243).

	*n*	% or mean (SE)
Sociodemographic factors		
Female gender	168	69.1
Age	243	42.3 (0.7)
Education		
Primary, lower secondary	67	27.6
Higher secondary	85	35.0
Higher professional, university	91	37.4
Not living with a partner	105	43.2
No job	65	26.7
Vulnerability factors		
Any abuse in childhood	127	53.1
Number of abuse in childhood	239	1.1 (0.1)
Any negative life event	153	64.0
Number of negative life events	239	1.2 (0.1)
Parental psychopathology^a^	110	45.3
Any somatic disorder	105	43.9
Number of comorbid somatic disorder	239	0.7 (0.1)
Physical Health Functioning (SF-36)^b^	243	75.4 (1.3)
Mental Health Functioning (SF-36)^b^	243	71.2 (1.3)
Neuroticism	239	5.7 (0.2)
Extroversion	239	6.8 (0.2)
Conscientiousness^a^	243	44.0 (0.4)
Clinical factors		
Age of onset of anxiety disorder (years)	243	17.7 (0.8)
Severe anxiety disorder^c,d^	52	21.4
Panic disorder^d^	51	21.0
Agoraphobia^d^	12	4.9
Social phobia^d^	141	58.0
Generalized anxiety disorder^d^	66	27.2
At least two anxiety disorders^d^	24	9.9
Any mood disorder		
Never	109	44.8
Remitted	58	23.9
Current	76	31.3
Any alcohol use disorder		
Never	203	83.5
Remitted	23	9.5
Current	17	7.0
Medication use^d^	55	23.0
Service use for mental health problems		
None	140	58.6
General medical care only	35	14.6
Mental health care	64	26.8

a Assessed at the second wave, T_1._

b This scale ranges from 0 (low functioning/ill health) up to until 100 (high functioning/good health).

c Severity based on the Sheehan Disability Scale.

d Twelve months.

**Table 3.** Putative predictors for intermittent course or chronic course of the anxiety disorder in the general population (*n* = 243), compared with a remitted course and adjusted for age and gender.

	Intermittent (*n* = 34)		Chronic (*n* = 20)		
	RRR	95% CI	RRR	95% CI	Chronic vs Intermittent *p* < 0.05
Sociodemographic factors					
Gender	2.35	0.93–5.99	1.18	0.43 -3.22	
Age/SD^a^	0.92	0.64–1.33	0.90	0.57–1.42	
Education					
Primary, lower secondary	Ref.		Ref.		
Higher secondary	1.18	0.41–3.44	0.68	0.21–2.16	
Higher professional, university	**2.50** *	**1.01–6.20**	0.57	0.18–1.76	
Not living with a partner	0.56	0.25 -1.26	**5.69** **	**1.81 -17.94**	*
No job	0.92	0.39 -2.13	1.20	0.43 -3.33	
Vulnerability factors					
Any abuse in childhood	**2.76** *	**1.21–6.29**	1.57	0.61–4.02	
Number of childhood abuses/SD^a^	1.30	0.91–1.85	1.32	0.85–2.06	
Any negative life event	0.82	0.38–1.77	1.74	0.60–5.04	
Number of negative life events/SD^a^	1.07	0.73–1.56	**1.71** *	**1.14–2.58**	
Parents psychopathology^b^	1.09	0.52 -2.29	2.44	0.93 -6.45	
Any somatic disorder	1.01	0.46–2.23	0.87	0.32–2.35	
Number of comorbid somatic disorder/SD^a^	0.96	0.64–1.44	1.05	0.66–1.69	
Physical functioning SF36/SD^a^	0.86	0.60–1.23	0.68	0.45,1.02	
Mental functioning SF36/SD^a^	0.71	0.50–1.02	0.42***	**0.28–0.65**	
Neuroticism/SD^a^	**1.49** *	**1.01–2.19**	**1.91** *	**1.16,3.15**	
Extroversion/SD^a^	0.98	0.66–1.43	0.66	0.42,1.06	
Conscientiousness^b^/SD^a^	0.90	0.62–1.30	0.68	0.43,1.08	
Clinical factors					
Age of onset anxiety disorder/SD^a^	0.70	0.44–1.10	0.81	0.48,1.38	
Severe anxiety disorder^c^	1.34	0.55 -3.23	3.62**	**1.39 -9.41**	
Panic disorder^c^	0.30	0.09 -1.03	0.56	0.16 -2.01	
Agoraphobia^c^	1.87	0.46 -7.53	1.15	0.13 -9.79	
Social phobia^c^	2.05	0.92 -4.60	1.50	0.56 -3.99	
Generalized anxiety disorder^c^	1.16	0.51 -2.62	1.56	0.59 -4.17	
At least 2 anxiety disorders^c^	2.46	0.86 -7.07	3.02	0.87 -10.46	
Any mood disorder, ref. is never					
Remitted	1.54	0.60–3.98	2.75	0.78–9.74	
Current	1.47	0.62–3.48	3.05	0.96–9.64	
Any alcohol use disorder, ref. is never					
Remitted	1.29	0.34–4.96	1.22	0.24–6.11	
Current	1.17	0.24–5.78	1.65	0.32–8.51	
Medication use^c^	1.71	0.74 -3.95	**3.49** *	**1.33 -9.16**	
Service use for mental health problems					
General medical care only	**2.69** *	**1.05–6.89**	2.14	0.51–8.92	
Mental health care	1.25	0.50–3.15	**3.61** *	**1.29–10.06**	

Bold: Significant RRR at the 0.05 level, 2-sided test. RRR: relative risk ratio; CI: confidence interval; Ref: reference category.

a Per standard deviation (SD) increase: SD age = 11.3; SD neuroticism = 3.1; SD extraversion = 3.3; SD conscientiousness = 6.4; SD number of childhood abuses = 1.3; SD number of negative life events = 1.2; SD number of somatic disorders = 1.2; SD mental functioning = 20.7; SD physical functioning = 20.7; SD age of onset anxiety disorder = 13.1.

b Assessed at the second wave, T_1._

c Twelve months.

* *p* < .05. **p < .01. ***p < .001.

## Results

### Characteristics of the study cohort

The characteristics of the study sample, 243 subjects with a 12-month anxiety disorder at baseline, are presented in Table 2. Twenty-one percent (*n* = 51) met criteria for a panic disorder, 4.9% (*n* = 12) for agoraphobia, 58.0% (*n* = 141) for social phobia, 27.2% (*n* = 66) for GAD, and 9.9% (*n* = 24) for two or more anxiety disorders. The majority of subjects (58.6%) did not make any use of services for their mental health problems, 14.6% made use of general medical care and the remaining 26.8% made use of mental health care.

### Course

Of 243 subjects, a majority of 77.8% (*n* = 189) achieved remission during follow-up. Of these, 82.5% achieved remission at T1, whereas 17.5% achieved remission at T_2_. Fourteen percent (*n* = 34) had an intermittent course and 8.2% (*n* = 20) had a chronic course trajectory.

### Subthreshold symptoms and transition

Of the remitting group (*n* = 189), 53.4% (*n* = 101) did not have any residual symptoms at T_2_, and 46.6% (*n* = 88) had subthreshold symptoms.

Of this group of 189 subjects, 15 (7.9%) made a transition to another diagnostic group, 20 (10.6%) still had a comorbid disorder and 154 (81.5%) were free of any diagnosis at T_2._ Taking both subthreshold symptoms and transitions to other diagnostics groups into account, only 52.4% (*n* = 99) of those in remission were free of anxiety symptoms and did not have another disorder. This translates to 40.7% of the total sample (99 of 243 subjects).

### Putative predictors of an intermittent and chronic course

The sociodemographic, vulnerability and clinical factors for the intermittent and chronic groups compared with the remission group are shown in Table 3. Predictors for an intermittent and chronic course were different. Of the sociodemographic factors, higher professional education was significantly associated with an intermittent course. Of the vulnerability factors, childhood abuse and neuroticism were significantly associated with an intermittent course. Of the clinical factors, only general medical care was significantly associated with an intermittent course.

More significant predictors were found for the chronic course trajectory than the intermittent group. Of the sociodemographic factors, not living with a partner was associated with chronic course. Of the vulnerability factors, number of negative life events was associated with chronic course, and, as in intermittent course, neuroticism was significantly associated. Of the clinical factors, severity of the anxiety disorder, medication use, lower mental functioning and mental health care use were all associated with a chronic course trajectory.

The predictor ‘living with a partner’ was the only predictor which differed significantly between the intermittent and chronic course groups.

## Discussion

This longitudinal study examined the 6-year course of anxiety disorders in the general population. It is an addition to the literature, since, to our knowledge, this is the first study conducted on the long-term course of anxiety disorders in the general population in which transition to residual subthreshold symptoms and transition to other diagnostic groups has been taken into account. We thereby prevent a too optimistic view on the course of anxiety disorders. Results showed that the majority of the subjects with anxiety disorders (77.8%) achieved remission within the 6-year follow-up. Results are in line with earlier general population studies, such as 77% remission in the elderly population at 6-year follow-up (Schuurmans et al., 2005) and 66.9% remission after 3-year follow-up (Henriksen et al., 2015). Our findings corroborate the view that the course of anxiety disorders is more favourable in the general population as compared with clinical-based populations, which generally report lower remission rates, such as 58.4 % remission at 2-year follow-up (Spinhoven et al., 2011) and 32–55% remission at 12-year follow-up (Bruce et al., 2005). This view is further supported by our finding that use of psychotropic medication and mental health care was significantly associated with chronic course.

When transition to another diagnostic group and to subthreshold symptoms was taken into account, this percentage decreased to 52.4% of the remitting group without subthreshold symptoms or other diagnosis. Thus, slightly more than half of the remitting group actually did well after 6 years. Although remission rates were high, 14.0% of subjects had an intermittent course trajectory, and 8.2% a chronic course trajectory. Hence, course was unfavourable in one out of every five subjects with an anxiety disorder at baseline. Moreover, not all remitted subjects had regained their mental health due to residual symptoms or diagnostic instability.

In the remitting sample of 189 subjects, it appeared that the majority of subjects had no residual symptoms. Still, nearly half of the subjects had still some symptoms. These figures are also in line with findings from other studies in the general population. For example, in a 3-year follow-up Nay et al. (2013) found 60.4% remission of panic disorder without residual symptoms vs 11.9% remission of panic disorder with subthreshold symptoms, while in a cohort with adolescents Wittchen et al. (2000) found substantial subsyndromal symptoms for all mental disorders, where only 15% of that population was not affected by at least some clinically relevant symptoms of mental disorders.

In addition to data on residual symptoms, transitions to other diagnostic categories may also provide some insight into genuine health gain. In the remitting sample, a transition to other diagnostic categories was present in 7.9% of the cases. In comparison, Rhebergen et al. (2011) found in the general population that 9.8% of the subjects with an anxiety disorder without depression at baseline fulfilled criteria for depression at 7-year follow-up. Also, Wittchen et al. (2000) found that 8.1–23.1% of subjects with agoraphobia or panic had another diagnosis at follow-up, but it is not clear to what extent these diagnoses were already present or newly arisen ones. In a predominant clinical sample, Penninx et al. (2011) found that of subjects with an anxiety disorder but no mood disorder at baseline, 7% developed a mood disorder after 2 years of follow-up. Also, in the same sample, a study with 4-year follow-up showed that over 30% of remitted anxiety disorder subjects developed a mood disorder (Scholten et al., 2016). These results suggest that transitions to other disorders occur less frequently in the general population as compared with clinical samples. This may imply that transitions, expressing diagnostic instability, are a characteristic of more severe anxiety disorders.

Our study showed clear predictors for the chronic course trajectory, whereas predictors for the intermittent trajectory were less clear. A strong association was found between not living with a partner and chronicity (estimated RRR 5.69). This is in line with previous studies (Batelaan et al., 2014; Rhebergen et al., 2011) and can be viewed in several ways. It may be that support of a partner is required to overcome anxiety, thus not living with a partner increasing the risk for chronicity. However, as course prior to baseline is unknown, it may also be that chronic anxiety hampers the ability to maintain a long-term relationship. In addition, not living with a partner may mediate the association between for example, childhood trauma or personality problems and chronic course. Due to limited statistical power we could not conduct detailed multivariable analyses to address these underlying mechanisms.

Furthermore, reduced mental functioning at baseline predicted chronicity, as was found previously (Batelaan et al., 2014; Spinhoven et al., 2016). While any negative life event was not associated with an unfavourable course, a higher number of negative life events resulted in a higher risk for a chronic course. One could imagine that coping fails during multiple life events, hampering the process of recovery. Of the clinical factors, severity, defined as self-reported impairment in at least two of four areas of role functioning assessed with the SDS, gave a higher risk for a chronic course, but was not significantly related to an intermittent course. Medication and mental health care use were also predictive of a chronic course. This was to be expected due to the naturalistic design, i.e. the most severe subjects are most likely to seek treatment. Contrary to our expectations, presence of multiple anxiety disorders and a comorbid mood disorder appeared not to be predictive. Previous research most often found these variables to be predictive (Batelaan et al., 2014; Penninx et al., 2011; Rhebergen et al., 2011). However, Kelly and Mezuk (2017) also found no association of remission of GAD comorbid with MDD.

Some limitations of this study should be taken into account. First, as in all longitudinal studies, there is attrition in the follow-up waves. Excluding participants who died during follow-up, the cumulative response rate was 70%. As attrition was not related to psychopathology at baseline (except for bipolar disorder but participants with bipolar disorder were already excluded for the present study), attrition is not likely to have biased our results. Second, although we examined course prospectively from baseline onwards, course prior to baseline is unknown. As a result, duration of an episode is unknown and could not be included as a predictor in this study. Duration has been found to be a predictor of course in earlier studies (Batelaan et al., 2014; Spinhoven et al., 2016). Third, in regard to life events, some recall bias might have happened, subjects with anxiety disorders might be more likely to remember negative life events, which could overestimate some of the associations. Fourth, predictors might vary between anxiety disorders. Due to insufficient sample size of specific anxiety disorders, it was not possible to conduct additional analyses per disorder. Fifth, the intermittent and chronic course groups were small, limiting statistical power. As a result, certain predictors may not have emerged from the data. This might explain why a positive family history, childhood trauma, multiple anxiety disorders and current mood disorder, which were predictive of course in previous clinical studies (Batelaan et al., 2014; Bruce et al., 2005; Penninx et al., 2011; Rhebergen et al., 2011), did not significantly predict unfavourable course trajectories in our study. On the other hand, some predictors we found could be due to chance, as the number of statistical analyses was quite large (Harrell et al., 1996; Streyerberg et al., 2000). This stresses the need for future studies on the course trajectories of anxiety disorders in the general population.

In conclusion, the course of anxiety disorders in the general population is rather favourable. However, 1 in 5 has an unfavourable course, and additionally, presence of subthreshold symptoms and transitions to other disorders impede mental health in those remitted from their former anxiety disorder. Predictors found in this study may contribute to the timely identification of people with an increased risk for chronicity.
